# Thermal properties and behavior of microencapsulated sugarcane wax phase change material

**DOI:** 10.1016/j.heliyon.2019.e02184

**Published:** 2019-08-19

**Authors:** Ekarat Tangsiriratana, Wanwisa Skolpap, Robert J. Patterson, Kobsak Sriprapha

**Affiliations:** a Department of Chemical Engineering, School of Engineering, Thammasat University, Pathumthani, 12120, Thailand; b School of Photovoltaic and Renewable Energy Engineering (SPREE), University of New South Wales (UNSW) Sydney, 2052, Australia; c Solar Energy Technology Laboratory, National Electronics and Computer Technology Center (NECTEC), 111 Thailand Science Park, Klong Luang, Pathumthani, 12120, Thailand; d Center of Clinical Engineering, School of Engineering, Thammasat University, Pathumthani, 12120, Thailand

**Keywords:** Chemical engineering, Phase change material, Sugarcane wax, Solar panel integration, Thermal conductivity, Thermal behavior

## Abstract

In this study, a micro-encapsulated phase change material (PCM) was composed of sugarcane wax−Al_2_O_3_composite as the core material and gelatin−gum Arabic as the polymer shell materials prepared by complex coacervation. The thermal behavior of solar panels integrated with this encapsulated PCM (EPCM) was investigated. The heat storage-dissipation performance and thermal stability of the sugarcane wax−based composite PCM layer with the heat capacity of 2.86 J/g·°C was influenced by its thickness. Increasing the composite PCM layer thickness from 4 mm to 7 mm could lower the module's front-facing glass temperature by 4% resulting in enhanced the photovoltaic power generation by 12% at the peak, because of the temperature storage ability of the composite PCM. Moreover, the thermal conductivity of the microencapsulated sugarcane wax was calculated using a steady-state one-dimensional energy balance equation. The thermal conductivities estimated across the composite PCM layer depth were found to be temperature dependent. A nonlinear regression of the power law thermal conductivity model gave a good agreement with the observed EPCM-surface temperatures.

## 1 Introduction

Conversion of solar energy into electricity directly using a photovoltaic (PV) is reduced due to reductions in the open circuit voltage as solar cell temperatures increase (Nada et al., 2018; Brinkworth et al., 1997). The operating temperature of a typical PV panel in full sun is generally around 60 °C, leading to reductions in the efficiency of approximately 0.5% per degree of temperature Celsius (Luque and Hegedus, 2003). As the scale of the deployment of photovoltaic electricity becomes larger this small percentage loss amounts to a significant amount of total energy lost. Methods to cool or increase the thermal mass of solar panels are of increasing importance. Numerous experimental investigations have been carried out to enhance solar panel efficiencies by decreasing their temperatures. Cooling has been attempted using heat exchange to water and forced convection from fans (Krauter, 2004; Amelia et al., 2016). Radiative cooling has recently been explored, making use of high PLQY materials, often nanomaterials (Zhao et al., 2019). An alternatively designed cooling system that is passive, such as pinning the temperature of the modules using a sufficient mass of a cheap, abundant and renewable PCM material with a solid to liquid phase change below the typical operating temperature of a solar panel, is very attractive.

A phase change material (PCM) employs the latent heat of the material as thermal storage with the benefit of greater storage density and “pinning” of the solar panel operating temperature. This is a potentially promising technology, to manage heat absorption or its release across a phase change in the material within a narrow temperature range (Waqas et al., 2018). The criteria of PCM selection depends on its melting point in the desired operating range to provide suitable heat exchange surface (Dutt Sharma et al., 2004). Among the various different types of PCM, organics such as sugarcane wax with a melting point of 77.6–80 °C (Lopes, 2010) are attractive since they are typically noncorrosive, abundant and inexpensive (Giro-Paloma et al., 2016). Furthermore, sugarcane wax can be a promising organic PCM and desirable to be applied in high surrounding temperature of tropical regions due to its high melting point. However, the drawbacks of the organic PCM have been limited thermal conductivity (typically between 0.2 and 0.7 W/m·K), and instability of PCM properties (Fan and Khodadadi, 2011). The thermal conductivity of composite materials can be estimated by experimental and theoretical approaches. The steady-state methods were applied to experimentally measure thermal conductivity using hot plate method (Pratt, 1962), heat flow meter, laser flash analyzer (Kinkelin et al., 2017), thermal conductivity analyzer (Lv et al., 2019). Moreover, the quasi steady-state model for estimating thermal conductivity of PCM was verified with experimental results (Kothari et al., 2019). Furthermore, the predictive effective thermal conductivity models of composite materials were developed using Maxwell model (Xu et al., 2016), Agari and Uno model (Agari et al., 1990), Russell model (Russell, 1935) and fractal theory (Zheng et al., 2019). These analytical models depend on composition and geometry of composite materials. The mechanical and thermal stability of microcapsules formed by the complex coacervation process can be improved by heating, desolvation, or crosslinking (Timilsena et al., 2016).

In previous research, a new design for low melting point organic PCMs containing metal nanoparticles to enhance the thermal conductivity has been suggested. Wang et al. (2014) reported that the addition of 1 wt% TiO_2_ nanoparticles in paraffin wax improves the latent heat capacity. Babapoor and Karimi (2015) studied the effect of different types of metal oxide nanoparticles such as SiO_2_, Al_2_O_3_, Fe_2_O_3_, and ZnO on the thermal energy storage properties of a paraffin-nanoparticle PCM. They found that the addition of Al_2_O_3_nanoparticles showed the greatest potential to improve the thermal energy capacity of nanocomposite PCMs. This was consistent with the study of Nada et al. (2018) about thermal efficiency of integrated PV module with parrafin wax (pure PCM) and parrafin wax/Al_2_O_3_. Comparing to the PV panel alone, the surface temperatures of PV−pure PCM and PV−parrafin enhanced with Al_2_O_3_ were lowered by 8.1 and 10.6 °C, respectively and the thermal efficiencies were improved by 5.7 and 13.2%, respectively. However, paraffin wax has competing applications in heating, lighting and lubrication, where sugarcane wax currently is perceived solely as a waste product. The performance of melting and solidification process and thermal conductivity of paraffin wax was greatly improved with nano−dispersion of Al_2_O_3_compared to that of CuO (Arasu et al., 2011). However, no study has yet quantified the thermal performance of Al_2_O_3_ nanoparticles in sugarcane wax, nor the thermal gradients present in the PCM integrated into a working PV panel. This knowledge is central to the design of functioning PCM-based module cooling layers in the field.

A technical difficulty that arises in the application of PCMs to PV panels is leakage of the melted PCM during the phase change process. Therefore, PCM encapsulation is an important problem and the encapsulant material must be carefully selected with a compatible fabrication method. Complex coacervation, an encapsulation technique, was first systematically studied by De Jong and Kruyt in 1929 (Jong and Kruyt, 1929). In the next century, the technique has been implemented in numerous fields, including buildings (Arce et al., 2012), textiles (Pause, 2019) and food sciences (Ye et al., 2018). In this work, complex coacervation technique was applied for the first time to fabricate encapsulated sugarcane wax with dispersion of Al_2_O_3_ nanoparticles as core material to enhance its thermal performance. The PCM was entrapped inside a shell using oppositely charged biopolymers, in our case, microcapsules formation using blends of positively charged gelatin and negatively charged gum Arabic. The process consisted of three steps as follows: (i) emulsification to form the biopolymer and core phase; (ii) coacervation of the shell and (iii) cross linking to harden the shell and improve its thermal and mechanical properties.

In this study, microencapsulated sugarcane wax containing Al_2_O_3_nanoparticlesas a core material with gelatin/arabic gum as the encapsulant was prepared by a novel complex coacervation technique. To the best of our knowledge, this is the first report of the use of abundant, non-toxic and inexpensive waste sugarcane wax as a PCM. Its thermal performance was investigated by integrating the PCM composite into 5 W PV panels with different EPCM layer thicknesses, providing data central to PV panel cooling layer in the field. The proposed one dimensional steady-state energy balance model was applied to estimate thermal conductivity of the composite PCM using simple electro-thermal measurements of integrated PV cell.

## 2 Material and methods

### 2.1 Materials

Sugarcane wax was provided by the national science and technology development agency (NSTDA), Thailand. Gelatin B, gum Arabic, and sodium dodecylsulphate (SDS) were purchased from Ajax Finechem Pty Ltd., Australia. Al_2_O_3_ nanoparticles sized less than 50 nm were purchased from Sigma-Aldrich, USA. Glutaraldehyde, a cross linking agent, was supplied from Sigma – Aldrich, USA. All of chemicals were of analytical grade and used without further purification.

### 2.2 Preparation of nanocomposite PCM

A total of 200 mL of sugarcane wax was melted using a water bath shaker (GLS AQUA 12, Grant Instruments, Cambridge, UK) whose temperature was maintained at about 100 °C above the melting point of sugarcane wax for 45 min. A mixture contained 2.4% (w/v) of Al_2_O_3_ nanoparticles and 0.6% (w/v) of sodium dodecyl sulphate (SDS) in the melted sugarcane wax. The resulting mixture was then homogenized for 75 min in the water bath controlled at 100 °C and then cooled to room temperature naturally to form uniform solid composite particles.

### 2.3 Encapsulation by the complex coacervation method

The complex coacervation procedure was adapted from the standard procedure of micro-encapsulation by Luzzi and Gerraughty (1967). First, 1 g of gum Arabic and 1 g of gelatin were separately dissolved in 100 mL of de-ionized water. The previously-prepared nanocomposite PCM, as a core material, was gradually added into 100 mL of an aqueous solution of gelatin (pH 6.5) while stirring at a temperature above the melting point of 12.6 g of the core material to form an oil-in-water emulsion. The resulting mixture was then gently added to a gum Arabic solution (pH 6.5) dropwise over 90 min while stirring to induce gelatin-gum Arabic coacervation. The mixture was adjusted to a pH of 4.5 by addition of 10% (w/v) acetic acid solution and mixed until coacervates were formed. Finally, the suspension was rapidly cooled to 8 °C and then 6 mL of glutaraldehyde was added as a crosslinking-agent at a pH of 9 using 10% (w/v) sodium hydroxide solution to improve adhesion of the coacervate and the solution was stirred continuously for 12 h.

### 2.4 Differential scanning calorimetry (DSC) analysis

The thermo-physical properties of the PCM, such as melting point and latent heat of fusion, were analyzed using a DSC (Mettler Toledo DSC, Module 1, Schwerzenbach, Switzerland). A 4 mg sample of the prepared PCM sample was heated from 30 °C to 100 °C, which is above its glass transition temperature, at a constant heating rate of 10 °C/min. After each heating cycle, the DSC pan was cooled down to 30 °C using liquid nitrogen prior to measuring new samples.

A specific heat capacity was further estimated using the DSC analysis as described in Eq. (1),

(1) $$c_{p}=\frac{\Delta y}{H_{r}m}$$

where$c_{p}$ is the specific heat capacity of the PCM (J/g·°C), $H_{r}$ is the heating rate used in DSC measurement (°C/sec), Δy is the difference between the y-axis intercept of the enthalpy-temperature baseline curve and the sample measurement curve (mW), and m is the sample mass (mg).

Encapsulation ratio (ER) efficiency (EE) of the prepared mass ratio of core in shell materials of 6.3:1 (w/w) were estimated by Eqs. (2) and (3), respectively (Fang et al., 2017).

(2) $$\text{ER}=\frac{\Delta H_{m,\text{EPCM}}}{\Delta H_{m,\;\text{core}}}\times 100\%$$

(3) $$\text{EE}=\frac{\Delta H_{m,\text{EPCM}}+\Delta H_{c,\text{EPCM}}}{\Delta H_{m,\;\text{core}}+\Delta H_{c,\;\text{core}}}\times 100\%$$

where $\Delta H_{m,\;\text{EPCM}}\;$ and $\Delta H_{c,\;\text{EPCM}}$ is melting and solidifying enthalpy of encapsulated sugarcane wax/nano-Al_2_O_3_ composite PCM (J/g), respectively. $\Delta H_{m,\;\mathrm{core}}$ and $\Delta H_{c,\;\mathrm{core}}$ is melting and solidifying enthalpy of sugarcane wax/nano-Al_2_O_3_ composite PCM (J/g), respectively.

### 2.5 Particle size distribution

The particle size distribution of microcapsules was analyzed using Mastersizer 3000 (Malvern Instruments, UK) with particle adsorption index of 1.51, particle reflective index of 1.36. Microcapsules were suspended in water during analysis.

### 2.6 Experimental setup

#### 2.6.1 Thermal behavior observation of prepared EPCM

**Fig. 1:**
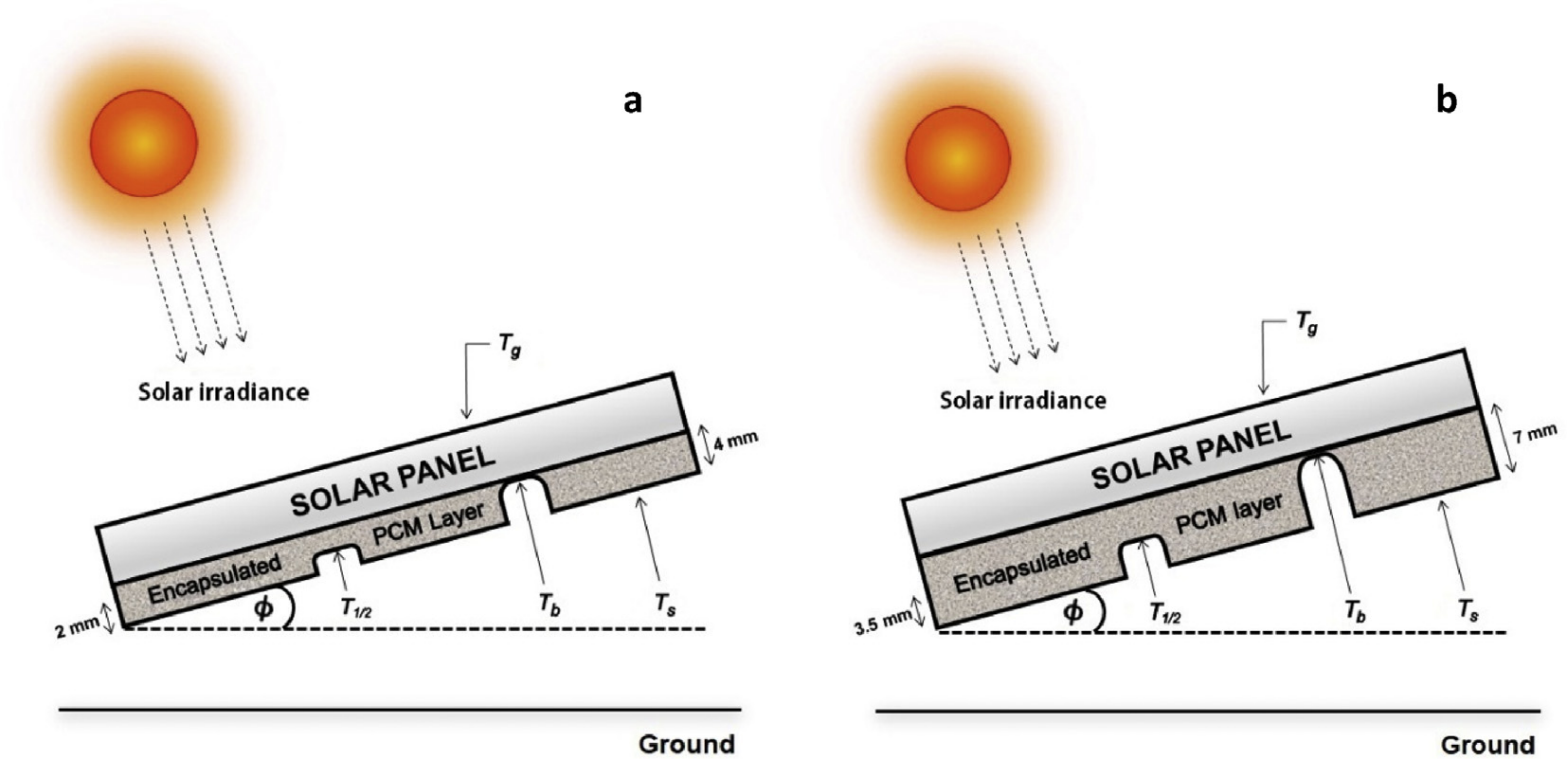
Experiment set up for recording thermal behavior of 4 mm−thick (a) and 7 mm−thick (b) EPCM layer. The $T_{i}$’s are the temperatures measured at various points in the structure.

A PV panel with poly-crystalline silicon cells (NUNSOLAR 5W-P) having a dimension of 270 × 200 × 17 mm was studied. The back side of each PV panel was coated with various thicknesses of the prepared EPCM layer from 0 to 7 mm as shown in Fig. 1. The measurements of solar irradiation, temperature and wind speed were taken from a height of 1.2 m above the ground and recorded every 10 min throughout 28 h starting on November 7, 2018 (day 1) at 13:10 until November 8, 2018 (day 2) at 17:00. At 510 W/m^2^ of average incident solar irradiation during 28 h observation, the average temperature of the panel was 35.1 °C ($T_{g}$) with the optimum PV module tilt angle (*ϕ*) of 15° to the horizontal for Thailand (Jacobson and Jadhav, 2018) and without wind across the panel. The temperature was measured using K type thermocouples, mounted along the depth of the EPCM layer. Three measurements were made for each sample thickness, one on the exposed back surface of the PCM ($T_{s}$), one in the center of the layer ($T_{1/2}$) and one at the interface between the PCM and the back surface of the PV panel ($T_{b}$). The signals generated from the thermocouples were recorded by Agilent 34972A LXI Data Acquisition/Data Logger Switch Unit, USA. Global horizontal irradiance data was measured using Kipp&Zonen CMP11 pyranometer (Delft, the Netherlands).

#### 2.6.2 Thermal conductivity experiment of EPCM

**Fig. 2:**
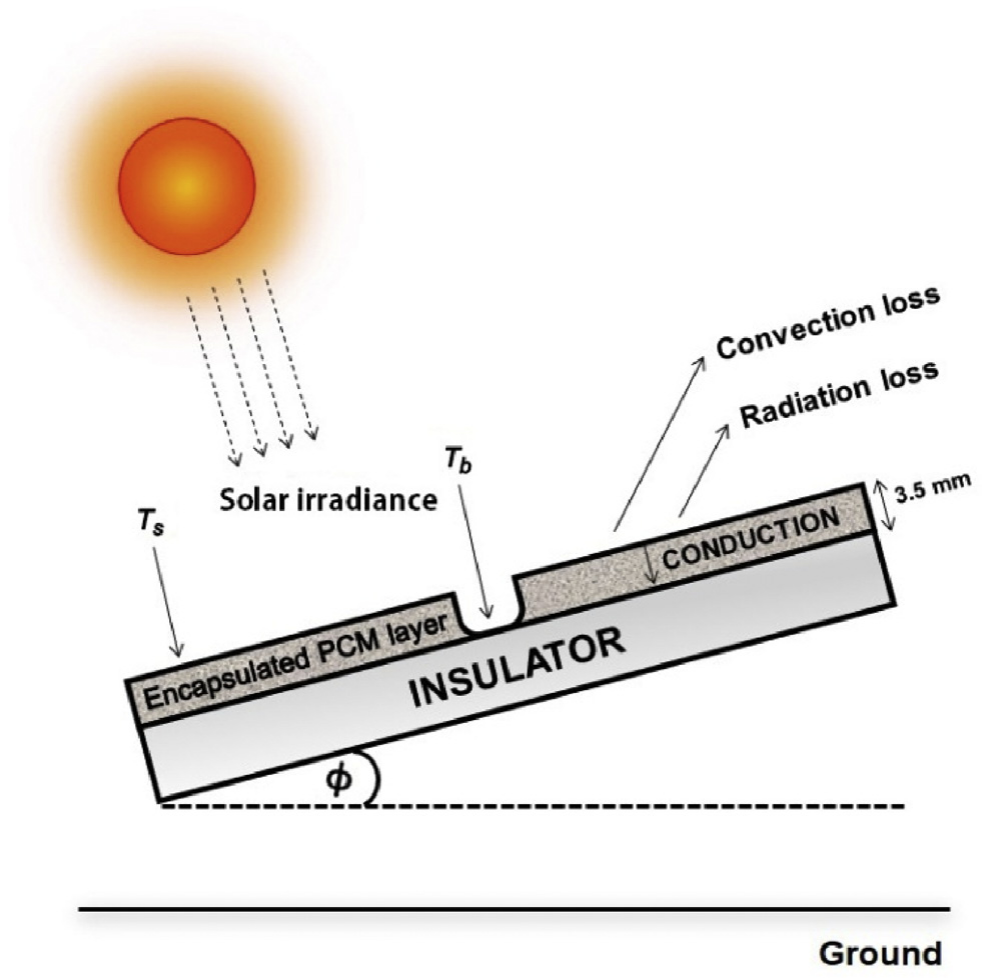
Inverted integrated PV cell for thermal conductivity calculation.

**Fig. 3:**
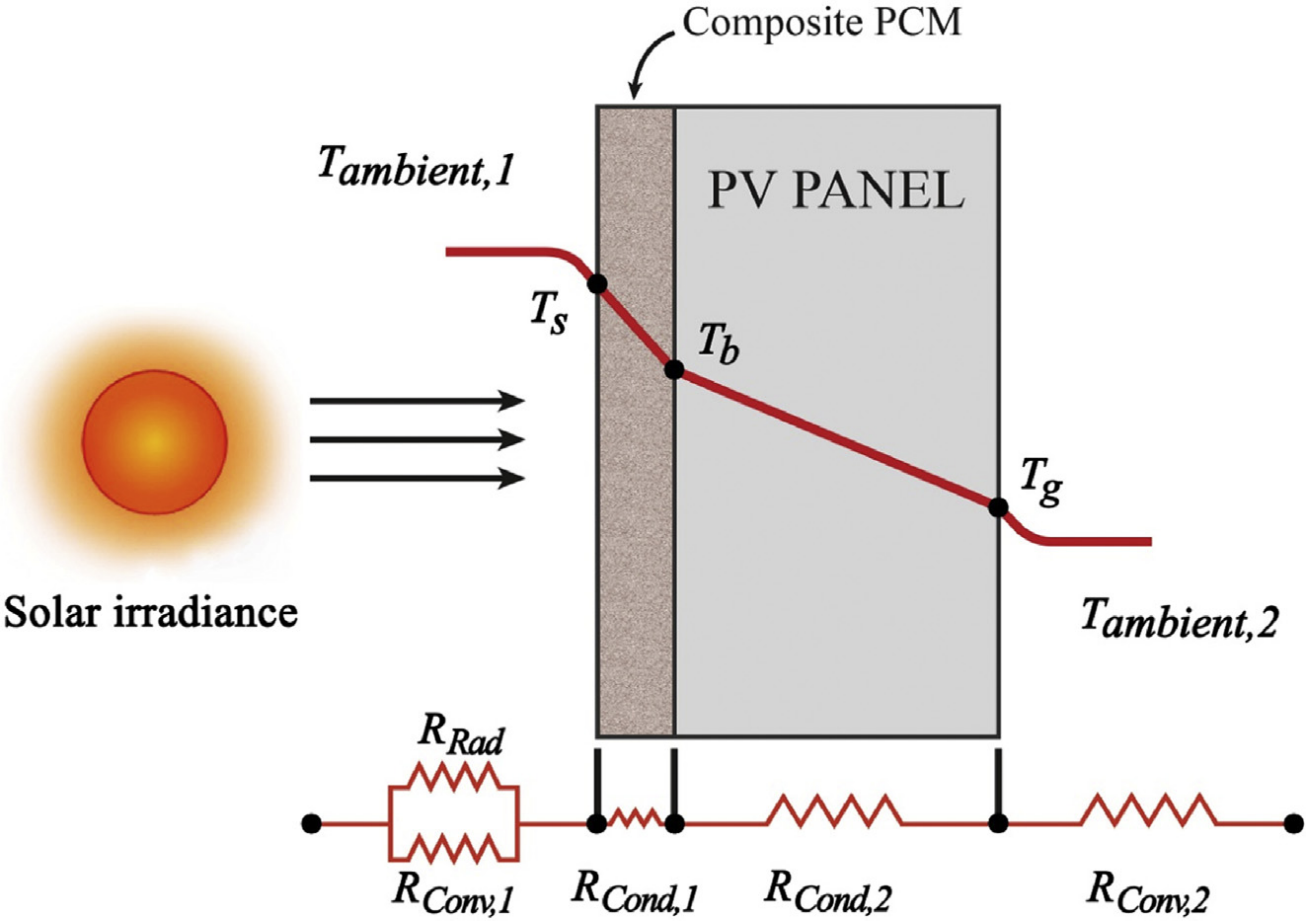
Thermal resistance diagram of the PCM on a PV panel.

A 3.5 mm sample of prepared EPCM layer situated in an integrated insulator for 15° tilt angle was the sun-facing side as shown in Fig. 2. The equivalent thermal resistance diagram for the PV system is depicted in Fig. 3.

### 2.7 Energy balance model

A steady-state one-dimensional energy balance model was developed to estimate the thermal conductivity of the prepared PCM without knowledge of the thermal properties of the PV cell materials using temperatures, output voltage and resistance measurements of integrated PV cell. Assumptions for the thermal model formulations are described as follows: (i) distribution of a temperature gradient in one-direction only; (ii) no power loss due to effect of shading via trees neighboring objects and any other means; (iii) negligible internal reflection between the layers of PV cell due to scattered light reflecting from the solar cell and (iv) the glass−coated solar panel allowing light to pass through with relatively complete blocking out thermal radiation (Ohsaki and Kokubu, 1999).

The steady-state 1-D energy balance is expressed as follows:

(4) $$E_{\text{in}}+E_{\text{sun}}=E_{\text{store}}$$

The total energy balance is represented by Eq. (4), where $E_{\text{in}}$ is the net thermal energy entering the EPCM layer (Fig. 2) through the process of conduction, convection and radiation as expressed in Eq. (5). $E_{\text{sun}}\;$ is the energy of total incident irradiation absorbed by the EPCM layer deposited on the backside of the PV cell exposed to sunlight as expressed in Eq. (18). $E_{\text{store}}\;$ is the energy stored in the EPCM layer due to its heat capacity as expressed in Eq. (19).

#### 2.7.1 The top surface energy balance

After sunlight passes through the atmosphere, it strikes the top surface of EPCM layer via conduction and portion of sunlight is transferred to the surroundings via convection and radiation.

(5) $$E_{\text{in}}=Q_{\text{cond}}+Q_{\text{conv}}+Q_{\text{rad}}$$

where$\;Q_{\text{cond}}$ is the conductive heat transfer through the EPCM layer (W), $Q_{\text{conv}}$ is due to the natural convective heat transfer to the surroundings (W), and $Q_{\text{rad}}$ is heat transferred outward at the top surface of the EPCM layer and then radiated into the surroundings (W).

##### 2.7.1.1 Conduction

The thermal conductivity of the EPCM layer was estimated using 1-D heat conduction model as expressed in Eq. (6).

(6) $$Q_{\text{cond}}=\frac{kA_{s}\left(T_{s}-T_{b}\right)}{\Delta x}$$

In Eq. (6), k is the thermal conductivity of the EPCM layer (W/K·m), $A_{s}$ is top surface area of the EPCM layer (m^2^), $T_{s}$ is the measured top surface temperature of EPCM layer (K), $T_{b}\;$ is temperature at the back side of EPCM (K), and x is the EPCM layer thickness (m).

##### 2.7.1.2 Convection

A general convective heat transfer is represented by Eq. (7).

(7) $$Q_{\text{conv}}=hA_{s}\left(T_{s}-T_{\text{ambient}}\right)$$

where h is a convective heat transfer coefficient (W/m^2^·K) depending on a plate inclination angle.

Under a measured average wind speed less than 0.1 m/s, the heat transfer through the upward-facing EPCM layer was characterized as free convection. To estimate h value using Eq. (8), the maximum Nusselt number was selected by comparing values calculated between $Nu_{HHT\;}$ (Rich, 1953) (Eq. (9) and $Nu_{CAC\;}$(Churchill and Chu, 1975) Eq. (10). Therefore, hwas determined as follows.

(8) $$h=k_{\text{air}}\cdot \max\left(\frac{Nu_{HHT}\left(Ra\left(Lc\right)\left|sin\theta \right|\right)}{Lc},\frac{Nu_{CAC}\left(Ra\left(L_{H}\right)\cdot cos\theta \right)}{L_{H}}\right)0{}^{\circ}\leq \theta \leq 90{}^{\circ}$$

(9) $${\text{where }Nu_{HHT}=\left(0.65+0.36Ra^{1/6}\right)}^{2}\;1<Ra<1.5\times 10^{9}$$

(10) $$Nu_{CAC}=0.68+\frac{0.67Ra^{1/4}}{{{[1+\left(0.492/Pr\right)}^{9/16}]}^{4/9}}\;0.1<Ra<10^{9}$$

(11) Ra=GrPr

(12) $$Gr=\frac{\beta \left(T_{s}-T_{\text{ambient}}\right)gL_{c}^{3}}{\nu^{2}}$$

(13) $$L_{C}=\frac{\text{Area}\;\text{of}\;\text{PV}\;\text{panel}}{\text{Perimeter}\;\text{of}\;\text{PV}\;\text{panel}}$$

(14) $$L_{H}=\text{Height}\;\text{of}\;\text{PV}\;\text{panel}$$

In Eqs. (8), (9), (10), (11), (12), (13), and (14), Nu,Gr,Pr,and Ra are corresponding the Nusselt number, Grashoff number, Prandtl number, and Rayleigh number,β is the volume expansion coefficient estimated by inverting the absolute surrounding temperature (K^−1^), gis gravity force constant (m/s^2^), $\;L_{C}\;$ and $L_{H}\;$ are the characteristic lengths (m) inclined θ(= 75°) from vertical and $k_{\text{air}},\;\nu$ and *Pr* are corresponding air thermal conductivity, air kinematic viscosity (m^2^/s) and air Prandtl number where air properties were estimated at film temperature between surface temperature and ambient temperature.

##### 2.7.1.3 Radiation

The radiated heat from the surface of the EPCM layer to the atmosphere was calculated by Eq. (15).

(15) $$\;Q_{\text{rad}}=\varepsilon F\sigma A_{s}\left(T_{s}^{4}-T_{sky}^{4}\right)$$

where

(16) $$F_{\text{EPCM}-\text{sky}}=\frac{1}{2}\left(1+\text{cos}\phi \right)$$

(17) $$T_{\text{sky}}=\left(0.037536\times \;T_{\text{ambient}}^{1.5}\right)+\left(0.32\times T_{\text{ambient}}\right)$$

ε is the estimated emissivity of the EPCM layer exposed to the solar irradiation on the surface tilt angle (ϕ) of 15° from horizontal, σ is Stefan–Boltzmann constant while *F* is a Fill factor evaluated by Eq. (16), *T*_ambient_ is ambient temperature and $T_{\text{sky}}$ is sky temperature evaluated by Eq. (17).

#### 2.7.2 Heat source

The total incident solar irradiation is partially converted to electricity due to the absorbance index of its front material layer; which in turn produces heat accumulating in the PV panel resulting in increasing its surface temperature. Thus, the absorbed solar radiation is given as:

(18) $$E_{\text{sun}}=\;\propto \cdot G\cdot A_{s}$$

where∝ is the estimated absorptivity of the EPCM layer, *G* is solar irradiation of the sun (W/m^2^), and $A_{s}$ is the top area of the EPCM layer (m^2^).

#### 2.7.3 Heat storage

The amount of thermal energy stored in a PCM depends on its specific heat capacity and latent heat. However, a melting point of the prepared PCM was 68 °C which was significantly higher than the surface temperature. Therefore, the latent heat was negligible in the governing equation for heat storage as expressed in Eq. (19).

(19) $$E_{\text{store}}=mc_{p}\left(T_{s,i}-T_{s,f}\right)$$

wherem is the mass of composite PCM (kg), $c_{p}$ is specific heat capacity of EPCM analyzed by DSC technique (kJ/kg·°C), and$\;T_{s,i}\;$ and $T_{s,f}\;$ are initial and final temperatures of the surface exposed to solar radiation (°C), respectively.

## 3 Results and discussion

### 3.1 Particle size analysis

A multimodal particle size distribution of the prepared composite PCM ranged from 48.2−264 μm with an intermediate diameter of 131 μm as shown in Table 1. This particle size was more than 2−fold larger than the size distribution of melamine−formaldehyde microcapsule containing *n*−octadecane with nano−SiO_2_ hydrogel as surfactant ranged from 10 to 90 μm depending on the level of ammonium chloride use as a nucleating agent (Su et al., 2018). The smaller capsules causes high surface area−to−volume ratio contributing to improved thermal conductivity and latent heat stored energy (Oya et al., 2013).

**Table 1:** Particle size distribution of the prepared composite PCM.

Sample	Diameter (μm)		
	*d* (0.1)^a^	*d* (0.5)^b^	*d* (0.9)^c^
Sugarcane wax/nano-Al_2_O_3_ composite PCM	48.2	131	264

The particle size distribution for the composite PCM is separated into two halves.

a 10% of the population lies below the *d* (0.1) value.

b 50% of the population lies below the *d* (0.5) value.

c 90% of the population, the most common value of the particle size distribution, lies below the *d* (0.9) value.

### 3.2 Thermal properties

**Fig. 4:**
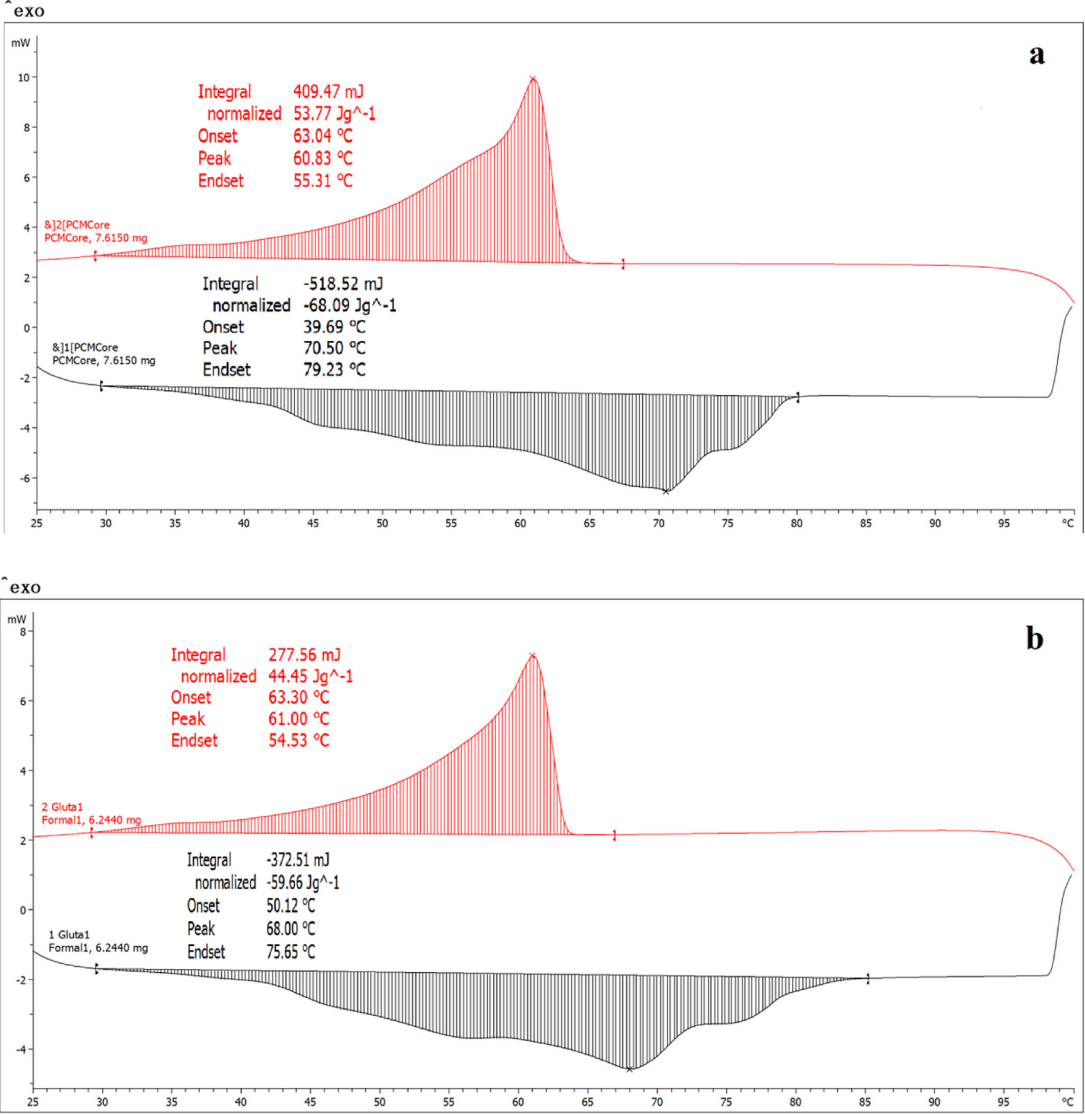
DSC thermograms of core (a) and EPCM (b).

DSC analysis was used to characterize the phase change behavior of the sugarcane wax/nano-Al_2_O_3_ composite PCM (core) and EPCM as shown in Fig. 4 (a) and Fig. 4(b), respectively. It was noticed that there is an endothermic peak during melting process and an exothermic peak during crystallization process.

The melting and solidifying (freezing) temperature and latent heat of fusion of composite PCM analyzed by DSC are summarized in Table 2.

**Table 2:** Thermal properties of the prepared sugarcane wax/nano-Al_2_O_3_ composite PCM, as well as the encapsulation ratio (ER) and encapsulation efficiency (EE).

Sample	Melting				Solidifying				ER∗ (%)	EE∗∗ (%)
	Onset temp (°C)	Endset temp (°C)	Specific enthalpy (J/g)	Peak temp (°C)	Onset temp (°C)	Endset temp (°C)	Specific enthalpy (J/g)	Peak temp (°C)		
Core	39.69	79.23	68.09	70.70	63.04	55.31	53.77	60.83	100	100
EPCM	50.12	75.65	59.66	68.00	63.30	54.53	44.45	61.00	87.6	85.4

Core is sugarcane wax/nano-Al_2_O_3_ composite PCM.

EPCM is encapsulated sugarcane wax/nano-Al_2_O_3_ composite PCM.

∗ ER was calculated using Eq. (2).

∗∗ EE was calculated using Eq. (3).

The onset and peak melting temperatures of core were observed at 39.69 °C and 79.23 °C, respectively with a latent heat of fusion of 68.09 J/g. The onset and peak melting temperatures of EPCM were observed at 50.12 °C and 68 °C, respectively with a latent heat of fusion of 59.66 J/g. The onset and peak solidifying temperatures of EPCM were observed at 63.30 °C and 61 °C, respectively with a latent heat of fusion of 44.45 J/g. After it was encapsulated, the latent heat of fusion and the melting temperature were reduced by 12.4% and 3.8%, respectively. The decreased melting temperatures of EPCM was caused by interface interaction between core and shell material (Pan et al., 2013) and fabrication of shape−stabilized shell surrounding the core (Li et al., 2013). The encapsulation ratio (ER) of EPCM, the effective encapsulation of core materials incorporated into the microcapsules, was 87.6% which is slightly higher than highest ER of 85% obtained from the microcapsules consisting of organic phase change as core material and polymethylmethacrylate as the shell (Han et al., 2017). The encapsulation efficiency (EE) of EPCM, the effective encapsulation of core materials incorporated into the microcapsules, was 85.4% which is comparable to the highest encapsulated beeswax/AgBr micro/nanocapsules of 83% (Yuan et al., 2019). The complete encapsulation of sugarcane wax/nano-Al_2_O_3_ into PCM microcapsules can be confirmed by DSC measurement of endothermic peak (61 °C) appeared near temperature of phase change of the core material (60.83 °C).

### 3.3 Thermal behavior

**Fig. 5:**
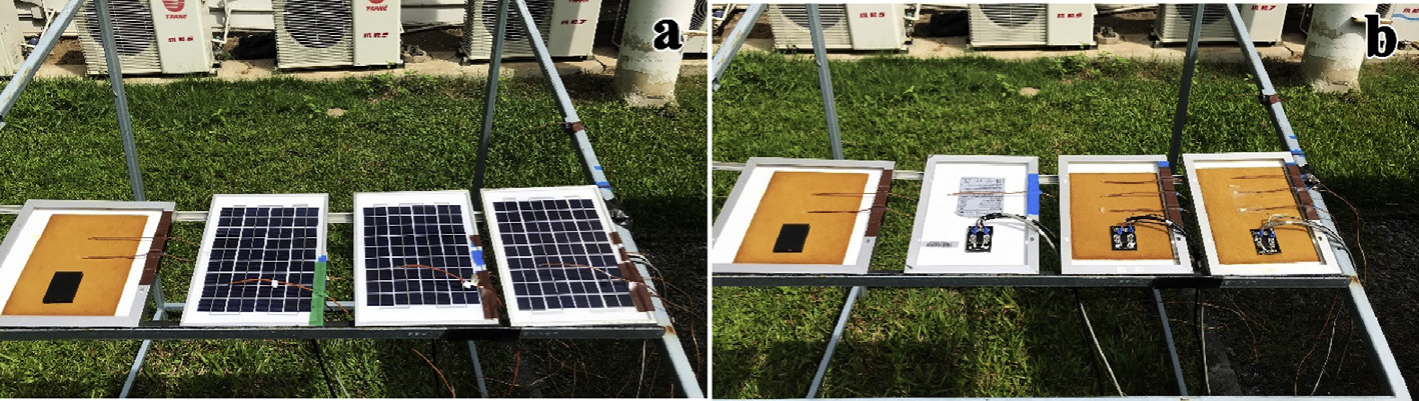
Experimental setup and thermocouples locations of PV modules in Pathumthani, Thailand (a) front side (b) backside.

**Fig. 6:**
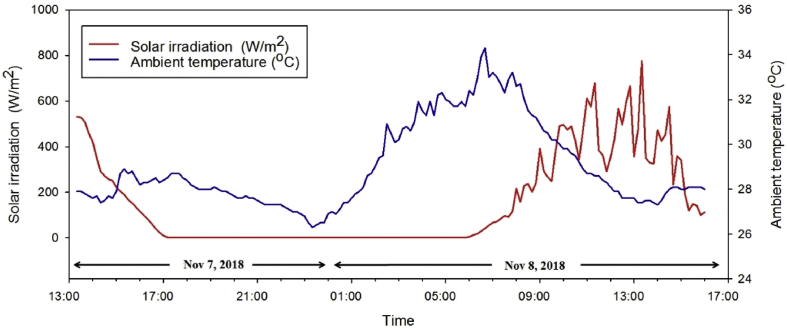
Incident solar irradiation data recorded in Pathumthani, Thailand during Nov 7–8, 2018.

**Fig. 7:**
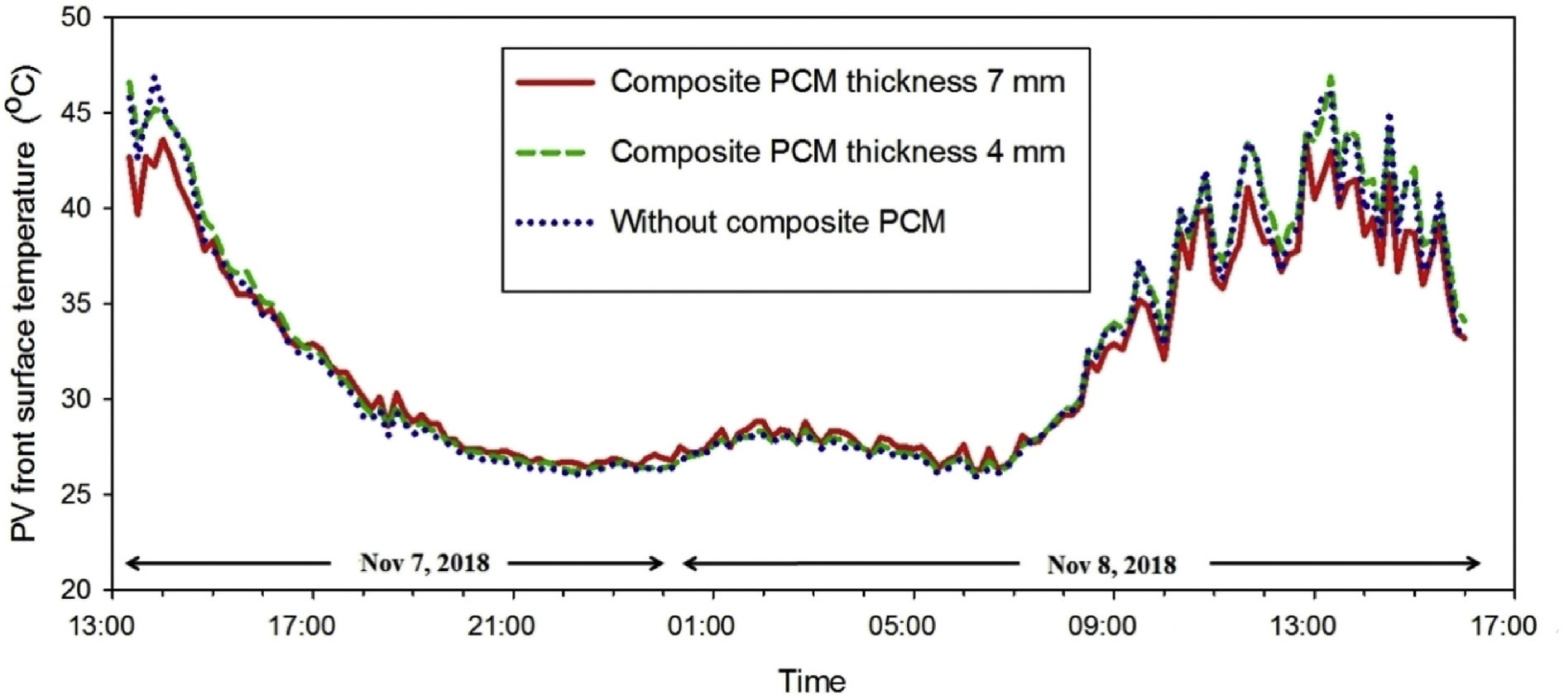
Profiles of PV front surface temperature measured in Pathumthani, Thailand during Nov 7–8, 2018. •; 7 mm−thick composite PCM layer, 4 mm−thick composite PCM layer, 0 mm−thick composite PCM layer (reference).

**Fig. 8:**
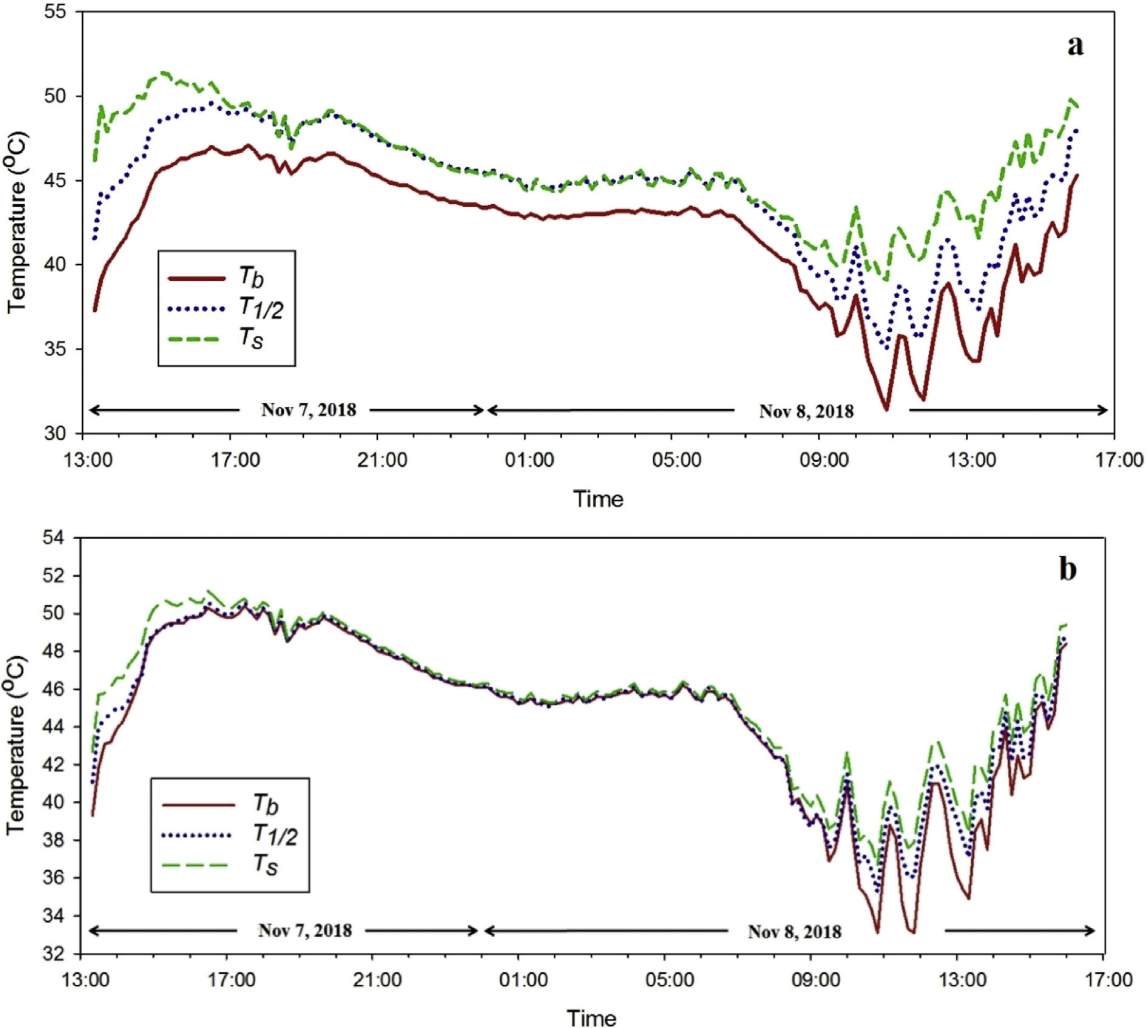
Profiles of temperature measured in Pathumthani, Thailand during Nov 7–8, 2018 for 7 mm (a) and 4 mm (b) thick EPCM layers.

In the practical application of developed PCM thermal energy storage, its thermal behavior of the reference PV cell (without composite PCM layer), that of 4 mm−, and 7 mm− thick composite PCM layer on the back side of the PV cell is investigated as illustrated in Fig. 5. At the first day of the thermal behavior experiment, the peak solar irradiation of 521 W/m^2^ at 13:20 decreased to zero at 17:20 while the solar irradiation reached the peak of 776 W/m^2^ at 13:20 on the second day as shown in Fig. 6. The reduction of the solar irradiation of day one caused the decline of the PV front surface temperature without composite PCM from about 45.8 °C to 32.2 °C while the PV front surface temperature of the 4 mm− and 7mm−thick EPCM layer was decreased from 45.2 °C to 32.7°C and from 42.2 °C to 32.9 °C, respectively as shown in the Fig. 7. Comparing between two different thicknesses, the outer surface temperatures of 7 mm− and that of 4 mm−thick EPCM layer was increased from 43 °C and reached a peak of 50°Cand from 46 °C and reached a peak of 52 °C, respectively as shown in Fig. 8(a). Similarly, the temperatures of the backside (0 mm) and the mid-depth (3.5 mm) of the 7 mm−thick EPCM layer were increased from 38 °C and reached a peak of 46 °C and from 42 °C and reached a peak of 48 °C, respectively as shown in Fig. 8(b). For the 4 mm− thick EPCM layer, the temperatures of the backside (0 mm) and the mid-depth (2 mm) were increased from 38 °C to 49 °C. This trend was attributed to solar radiation having been transferred and accumulated in the EPCM layer, causing it to melt. While melting, the EPCM layer stores thermal energy in the latent heat of its phase change, with the increase of temperatures of EPCM layer. It was observed that the thicker EPCM layer contributed to larger heat storage (Al Siyabi et al., 2018). Moreover, the front panel surface temperatures of reference PV and that of the EPCM layer deposition were dropped to about 26–27 °C without sunshine as shown in Fig. 7. The temperatures of the outer surface (7 mm) and that of the mid-depth (3.5 mm) of 7 mm thick EPCM layer were decreased from about 49 °C to 45 °C while its backside temperature was decreased from 46.6 °C to 43.4 °C, respectively as shown in Fig. 8 (a). For the 4 mm−thick EPCM layer, the temperatures were decreased from about 50 °C to 46 °C at all depth as shown in Fig. 8(b). When the outer surface temperature of EPCM layer decreased, it releases the stored latent heat energy causing solidification of the EPCM layer.

Table 3 illustrates the influence of front and back surface temperatures of the integrated PV panels of 0 mm− (reference PV panel), 4 mm−, and 7 mm−thick composite PCM layer on photovoltaic power output in sunshine period during 28 h observation.

**Table 3:** Effect of panel surface temperature on photovoltaic power generation of the integrated solar panels in sunshine hours during 28 h observation.

Composite PCM thickness	Sunshine duration		Power output	PV front surface temperature	Backside temperature
(mm)			(W)	(°C)	(°C)
0 (reference)	10:20–13:20	max	1.84	48.6	54.1
		min	1.11	45.0	48.6
		average	1.51	41.9	50.0
		S.D.	0.20	1.3	4.0
4	10:20–13:20	max	1.59	46.0	35.0
		min	0.97	40.8	27.5
		average	0.20	44.4	33.0
		S.D.	1.10	2.0	3.4
7	10:20–13:20	max	1.99	45.2	36.2
		min	1.21	38.2	30.7
		average	1.61	42.0	34.5
		S.D.	0.20	2.2	2.3
0 (reference)	14:20–16:40	max	0.63	45.0	49.0
		min	0.02	30.6	30.3
		average	0.18	36.5	37.5
		S.D.	0.17	6.0	7.8
4	14:20–16:40	max	0.60	43.9	45.0
		min	0.02	33.1	50.1
		average	0.17	37.4	48.8
		S.D.	0.17	3.2	1.6
7	14:20–16:40	max	0.71	41.2	42.4
		min	0.02	32.8	46.8
		average	0.20	36.4	45.5
		S.D.	0.20	2.5	1.4

Photovoltaic power output was calculated using Eq. (20).

(20) $$P=\frac{V^{2}}{R}$$

whereP is a power output of the PV panel (W), V is its voltage output (Volt)*,* and R is its electrical resistance (Ohm).

To understand the charge-discharge of EPCM on the back side of PV panel, front surface and back side temperatures and power output were observed. In sunshine duration during 10:20–13:20, the PV front surface temperature of the reference PV was lower than its back surface temperature while those of the PV modules integrated with composite PCM layer were higher than their back surface temperatures. The average photovoltaic power generation of the PV modules integrated with composite PCM layer was improved by 7%. The average PV cell temperature reduction of 2.3 °C was achieved with elevating composite PCM layer thickness from 4 to 7 mm. This is attributed that the composite PCM absorbs heat from the integrated PV cell and stores it as a latent heat without temperature increased during early melting stage of PCM (Khanna et al., 2018). It was suggested that low thermal conductivity and low melting point PCMs requires less time to complete melting and greater amount to lower PV temperature; which in turn provide better heat transfer performance under low PV operating temperature (Hassan et al., 2010; Khanna et al., 2018). After nearly complete melting the composite PCM in latent heat absorption period during 14:20–16:40, it resulted in excessive solar energy stored as a sensible heat (Khanna et al., 2018). Therefore, the backside temperatures started rising up between14:20 and 16:40 while the maximum photovoltaic power generated by the PV−7 mm EPCM layer module was about 12% higher than the PV without EPCM. This efficiency improvement is compatible with the application of PV−paraffin wax/Al_2_O_3_ achieved 13.2% efficiency increase comparing to PV module without PCM (Nada et al., 2018). However, the photovoltaic power generated by the PV−4 mm EPCM layer module was lower than the PV without EPCM throughout the sunshine period indicating inadequate thermal storage capacity.

### 3.4 *In situ* thermal performance of the composite PCM

Multiple melting/solidifying cycles of the prepared PCM during long−term operation is requirement to verify the long−term stability and durability for at least 5,000 cycles (Maxa et al., 2017). Thus, the thermal stability analyses of the composite PCM to store latent heat continued from Nov 8, 2018 for 14 consecutive days or 14 charging/discharging cycles. The *in situ* measurements in melting process of the composite PCM were investigated during Nov 9–22, 2018 as illustrated in Table 4. The panel efficiency was calculated using Eq. (21) (Mousavi Baygi and Sadrameli, 2018).

(21) $$\eta =\frac{\text{Maximum}\;\text{power}\;\text{with}\;\text{EPCM}-\text{Maximum}\;\text{power}\;\text{without}\;\text{EPCM}}{\text{Maximum}\;\text{power}\;\text{without}\;\text{EPCM}}$$

where η is the efficiency of the PV panel (%).

**Table 4:** Long-term thermal energy−storing performance of the composite PCM.

Composite PCM thickness	Sunshine duration		Solar irradiance	$\eta^{*}$	PV front surface temperature	Backside temperature
(mm)			(W/m^2^)	(%)	(°C)	(°C)
	Nov 9–15, 2018					
0 (reference)	10:20–13:20	max	302.19	reference	55.44	59.00
		min	163.89		42.87	45.08
		average	241.93		44.75	46.93
		S.D.	70.84		4.49	5.62
4	10:20–13:20	max	302.19	neg.	52.64	57.66
		min	163.89		40.37	45.20
		average	241.93		42.92	47.12
		S.D.	70.84		4.24	4.49
7	10:20–13:20	max	302.19	4.6	56.83	59.38
		min	163.89		44.06	44.68
		average	241.93		46.07	47.22
		S.D.	70.84		4.59	5.99
0 (reference)	14:20–16:40	max	402.53	reference	51.47	54.99
		min	198.78		34.98	36.05
		average	304.42		44.39	46.73
		S.D.	102.08		4.37	5.71
4	14:20–16:40	max	402.53	9.6	47.73	53.35
		min	198.78		33.62	35.82
		average	304.42		41.82	45.86
		S.D.	102.08		4.07	5.47
7	14:20–16:40	max	402.53	11.8	53.91	54.98
		min	198.78		35.36	35.10
		average.	304.42		45.78	45.68
		S.D.	102.08		4.98	5.96
	Nov 16–22, 2018					
0 (reference)	10:20–13:20	max	673.80	reference	45.83	46.64
		min	532.86		43.90	45.61
		average	618.19		44.10	46.53
		S.D.	75.02		2.48	3.62
4	10:20–13:20	max	673.80	0.2	49.83	43.01
		min	532.86		38.03	31.59
		average	618.19		49.03	41.08
		S.D.	75.02		5.74	6.78
7	10:20–13:20	max	673.80	neg.	48.43	50.60
		min	532.86		46.04	48.23
		average	618.19		40.23	44.64
		S.D.	75.02		7.14	5.73
0 (reference)	14:20–16:40	max	469.11	reference	36.55	62.30
		min	181.19		30.53	52.83
		average	322.55		36.35	55.13
		S.D.	144.08		7.40	5.81
4	14:20–16:40	max	469.11	neg.	66.49	61.82
		min	181.19		52.80	51.80
		average	322.55		59.19	55.38
		S.D.	144.08		6.38	4.37
7	14:20–16:40	max	469.11	5.8	62.28	66.00
		min	181.19		52.19	50.44
		average	322.55		55.46	58.42
		S.D.	144.08		6.01	4.45

neg. refers to a negative estimate.

$\eta^{*}$was calculated using Eq. (21).

After 9^th^ day of thermal performance (Nov 15, 2018) between 10:20 and 13:20, the efficiency of the panel with 7 mm thick EPCM layer was increased to 4.6% while that of the panel with 4 mm thick EPCM layer was estimated as a negative value. This indicates a hindered maximum output power of the 4 mm thick EPCM layer due to insufficient heat storage capacity within thinner composite layer thickness. In nearly complete melting period between 14:20 and 16:40, the efficiencies of the panel with 4 mm and 7 mm thick EPCM layers were increased to 9.6% and 11.8%, respectively. Reduction of the PV front surface temperatures were observed for all PV panel resulting in improvement of the panel efficiency (Chikate et al., 2015). After 16 charging/discharging cycles (Nov 22, 2018) at peak solar irradiation, the front surface temperatures of all PV modules were increased leading to insignificant efficiency improvement of the panels with PCM application. At lower solar irradiation during 14:20–16:40, the average front surface of the panel with 7 mm thick EPCM layer was reduced by 8% causing the 5.8% improvement of the panel efficiency. Conversely, the average front surface of the panel with4 mm thick EPCM layer was increased about 7% resulting negative value of panel efficiency. This indicates thermal instability for 4 mm composite PCM thickness after day 16 under full weather conditions. A possible solution to enhance PCM stability and performance is selection of suitable shell materials, i.e., hydrophilic, hydrophobic, and amphiphilic and the structure of the composite EPCM (Shchukina et al., 2018). The 7 mm composite PCM thickness improved the thermal stability of the PV module after 16^th^ day of application which was comparable to the optimum thickness of 5.26 mm reported in the study of Mourid and Alami (2018). Evidently, the relatively large microcapsules could enhance their heat storage capacity and thermal stability with the increase of EPCM thickness layer. Comparing to the 16^th^ cycles of climate testing of cycling durability of the prepared composite PCM, the thermal volume change in resistance of expanded clay−paraffin wax−geo−polymer composite material is observed after 60 day thermal cycling durability test under outdoors temperatures change of 23 °C–65 °C (Hassan et al., 2018).

### 3.5 Thermal conductivity estimation

**Fig. 9:**
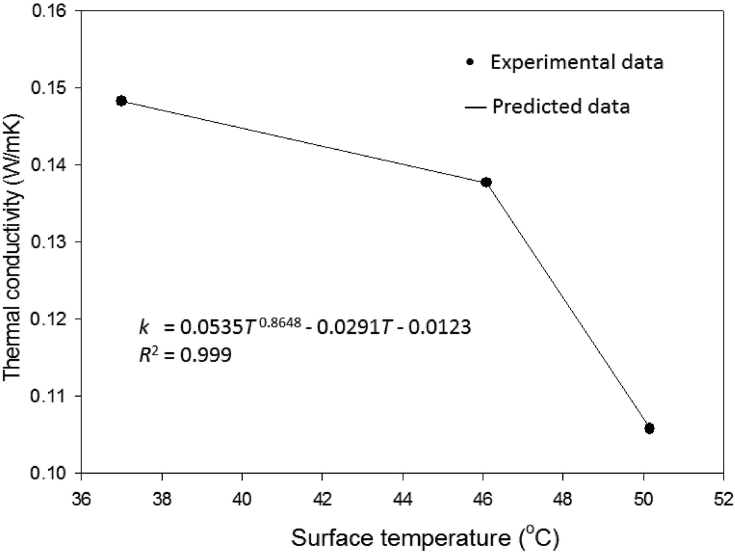
A nonlinear model of thermal conductivity of the sugarcane wax−based composite PCM.

The numerical result of thermal conductivity of the 3.5 mm-thick prepared EPCM layer was given by solving the steady-state 1-D energy balance described in Eq. (4). It was noted that a functional relationship between the calculated thermal conductivity of the prepared EPCM layer and temperature at any depth existed. This relationship was quantified using nonlinear regression to perform a functional fit to the data. The predicted thermal conductivity model was plotted against the temperature at any depth of the prepared EPCM layer as illustrated in Fig. 9. The temperature-dependent thermal conductivity function during melting period was depicted in Eq. (22).

(22) $$k=0.0535T^{0.8648}-0.0291T-0.0123$$

wherek is the thermal conductivity of composite PCM layer (W/K·m) and T is temperature at any depth of the composite PCM layer (°C). A good agreement between predicted and observed values of thermal conductivities of the prepared EPCM layer was pronounced as shown in Fig. 9.

Adding metal oxides to PCMs can enhance the thermal conductivity. However, poor mixtures of particles to PCMs cause the increase of interfacial resistance and reduction of thermal storage performance resulting in no positive effect on the PCM (Teng and Yu, 2012). As shown in Table 5, at a loading of 4% Al_2_O_3_, ZnO, Fe_2_O_3_, and Si_2_O_3_, the measured thermal conductivity of paraffin-metal oxide composites were enhanced between 1.8 and 2.25 times (Babapoor and Karimi, 2015). For the Al_2_O_3_ loading of 3% and 4%, the thermal conductivity of the composite paraffin was increased with increasing Al_2_O_3_ loading. In this study, the estimated thermal conductivity of the EPCM layer was improved 1.63-fold over that of sugarcane wax.

**Table 5:** Comparison of thermal conductivity with various PCMs and material additives.

Reference	Organic PCM	Melting point of organic PCM (°C)	k of organic PCM (W/m·K)	Additive particle	Mass fraction of additive particle (%wt)	k of EPCM (W/m·K)	Improvement
This paper	Sugarcane wax	62 (Inarkar and Lele, 2012)	0.08 (El-Sayed and Mostafa, 2016)	Al_2_O_3_	3%	0.131 (estimated using Eq. (4))	1.63 times
Lin and Al-Kayiem (2016)	Paraffin wax	60	0.18	Cu	2%	0.320 (measured)	1.78 times
Babapoor and Karimi (2015)	Paraffin wax	53–57	0.40	Al_2_O_3_	4%	0.900 (measured)	2.25 times
	Paraffin wax	53–57	0.40	ZnO	4%	0.750 (measured	1.88 times
	Paraffin wax	53–57	0.40	Fe_2_O_3_	4%	0.730 (measured	1.83 times
	Paraffin wax	53–57	0.40	Si_2_O_3_	4%	0.720 (measured)	1.8 times
Sharma et al. (2016)	Palmitic acid	60–62	0.20	TiO_2_	3%	0.280 (measured)	1.4 times
Chaichan et al. (2017)	Paraffin wax	45	0.058	Al_2_O_3_	3%	0.091 (measured)	1.57 times

The predicted thermal conductivity by the model Eq. (22) was verified by comparing to the experimental values for the thermal conductivity given in Table 5.

## 4 Conclusion

The thickness of the encapsulated sugarcane wax as PCM situated in the back of integrated PV cell had a positive effect on its heat absorbing-dissipating performance and its thermal stability. The front-facing glass temperature could reduce by about 4% at the peak with the increase of EPCM layer thickness from 4 mm to 7 mm due to larger amount of heat stored. Consequently, the average photovoltaic power generated was increased by 7%. The calculated thermal conductivities along depths of the EPCM layer thickness using developed steady-state 1-D energy balance model were comparable with the reported result of analytical model. A well-fitted nonlinear regression model of temperature-dependent power law thermal conductivity during melting period gave good agreement with the observed values. The prepared EPCM suggests guideline for developing heat absorbing material to provide benefits of temperature reduction of front-facing glass of solar panel and enhancement of power generation.

## Declarations

### Author contribution statement

Ekarat Tangsiriratana: Performed the experiments; Wrote the paper.

Wanwisa Skolpap, Robert Patterson: Analyzed and interpreted the data; Wrote the paper.

Kobsak Sriprapha: Contributed reagents, materials, analysis tools or data.

### Funding statement

This work was supported by the School of Engineering, Thammasat University, Thailand.

### Competing interest statement

The authors declare no conflict of interest.

### Additional information

No additional information is available for this paper.
